# 2-Cyano­anilinium bromide

**DOI:** 10.1107/S1600536809035223

**Published:** 2009-09-09

**Authors:** Li Zhang

**Affiliations:** a Ordered Matter Science Research Center, College of Chemistry and Chemical Engineering, Southeast University, Nanjing 210096, People’s Republic of China

## Abstract

In the cation of the title compound, C_7_H_7_N_2_^+^·Br^−^, the nitrile group and the benzene ring are almost coplanar (r.m.s. deviation = 0.0043 Å). In the crystal, the cations and anions are connected by inter­molecular N—H⋯Br hydrogen bonds, forming a two-dimensional network parallel to (010).

## Related literature

For nitrile derivatives, see: Fu *et al.* (2008 ▶); Wang *et al.* (2002 ▶). Nitrile derivatives used in the construction of novel metal-organic frameworks. For applications of metal-organic coordination compounds, see: Fu *et al.* (2007 ▶); Chen *et al.* (2000 ▶); Fu & Xiong (2008 ▶); Xiong *et al.* (1999 ▶); Xie *et al.* (2003 ▶); Zhang *et al.* (2001 ▶).
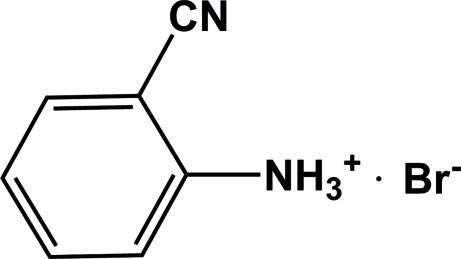


## Experimental

### 

#### Crystal data

C_7_H_7_N_2_^+^·Br^−^
*M* *_r_* = 199.06
Monoclinic, 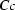

*a* = 5.7844 (12) Å
*b* = 15.896 (3) Å
*c* = 8.4882 (17) Å
β = 92.72 (3)°
*V* = 779.6 (3) Å^3^
*Z* = 4
Mo *K*α radiation
μ = 5.19 mm^−1^
*T* = 298 K
0.40 × 0.05 × 0.05 mm

#### Data collection

Rigaku Mercury2 diffractometer
Absorption correction: multi-scan (*CrystalClear*; Rigaku, 2005 ▶) *T*_min_ = 0.65, *T*_max_ = 0.77
3848 measured reflections
1773 independent reflections
1581 reflections with *I* > 2σ(*I*)
*R*_int_ = 0.034

#### Refinement

*R*[*F* ^2^ > 2σ(*F* ^2^)] = 0.025
*wR*(*F* ^2^) = 0.050
*S* = 0.87
1773 reflections
93 parameters
2 restraints
H-atom parameters constrained
Δρ_max_ = 0.47 e Å^−3^
Δρ_min_ = −0.41 e Å^−3^
Absolute structure: Flack (1983 ▶), 872 Friedels pairs
Flack parameter: 0.004 (13)

### 

Data collection: *CrystalClear* (Rigaku, 2005 ▶); cell refinement: *CrystalClear*; data reduction: *CrystalClear*; program(s) used to solve structure: *SHELXTL* (Sheldrick, 2008 ▶); program(s) used to refine structure: *SHELXTL*; molecular graphics: *SHELXTL*; software used to prepare material for publication: *SHELXTL*.

## Supplementary Material

Crystal structure: contains datablocks I, global. DOI: 10.1107/S1600536809035223/xu2593sup1.cif

Structure factors: contains datablocks I. DOI: 10.1107/S1600536809035223/xu2593Isup2.hkl

Additional supplementary materials:  crystallographic information; 3D view; checkCIF report

## Figures and Tables

**Table 1:** Hydrogen-bond geometry (Å, °)

*D*—H⋯*A*	*D*—H	H⋯*A*	*D*⋯*A*	*D*—H⋯*A*
N1—H1*A*⋯Br1	0.89	2.36	3.234 (3)	168
N1—H1*B*⋯Br1^i^	0.89	2.47	3.355 (3)	173
N1—H1*C*⋯Br1^ii^	0.89	2.42	3.286 (3)	164

Symmetry codes: (i) 
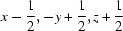
; (ii) 
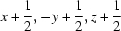
.

**Fig. 1.**
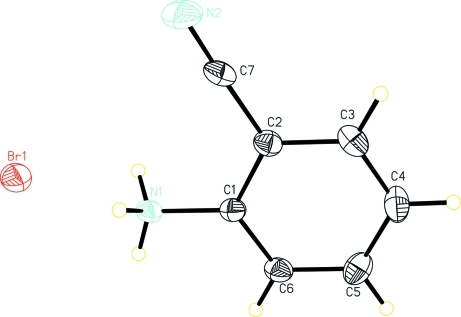
The structure of the title compound with the atomic numbering scheme. Displacement ellipsoids are drawn at the 30% probability level.

**Fig. 2.**
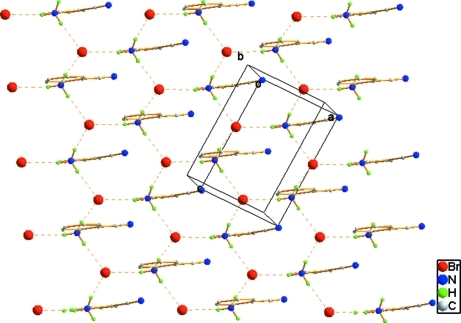
The crystal packing of the title compound, viewed along the b axis showing the N—H···Br hydrogen bonding (dashed lines). H atoms not involved in hydrogen bonding have been omitted for clarity.

## supplementary crystallographic information

### Comment

The construction of metal-organic coordination compounds has attracted much attention owing to potential functions, such as permittivity, fluorescence, magnetism and optical properties (Fu *et al.*, 2007; Chen *et al.*, 2000; Fu & Xiong (2008); Xie *et al.*, 2003; Zhang *et al.*, 2001; Xiong *et al.*, 1999). Nitrile derivatives are a class of excellent ligands for the construction of novel metal-organic frameworks (Wang *et al.*, 2002; Fu *et al.*, 2008). We report here the crystal structure of the title compound.

In the 2-cyanoanilinium cation (Fig.1), the nitrile group and the benzene ring are almost coplanar. The nitrile group C7≡N2 bond length of 1.142 (4) Å is within the normal range.

In the crystal structure, all the amine group H atoms are involved in N—H···Br hydrogen bonds (Table 1) with Br^-^ anions. These hydrogen bonds along with N—H···Br hydrogen bonds link the ionic units into a two-dimensional network (Fig. 2) parallel to the (0 1 0) plane.

### Experimental

The commercial 2-aminobenzonitrile (3 mmol, 0.55 g) and HBr (0.5 ml) were dissolved in ethanol (20 ml). Colourless needle-shaped crystals of the title compound suitable for X-ray analysis were obtained by slow evaporation at room temperature.

### Refinement

All H atoms attached to C and N atoms were positioned geometrically and treated as riding, with C—H = 0.93 Å, N—H = 0.89 Å and *U*_iso_(H) = 1.2U_eq_(C) and *U*_iso_(H) = 1.5U_eq_(N). A rotating-group model was used for the -NH_3_ group.

### Crystal data

**Table:**

C_7_H_7_N_2_^+^·Br^−^	*F*(000) = 392
*M**_r_* = 199.06	*D*_x_ = 1.696 Mg m^−^^3^
Monoclinic, *C**c*	Mo *K*α radiation, λ = 0.71073 Å
Hall symbol: C -2yc	Cell parameters from 1581 reflections
*a* = 5.7844 (12) Å	θ = 2.6–27.5°
*b* = 15.896 (3) Å	µ = 5.19 mm^−^^1^
*c* = 8.4882 (17) Å	*T* = 298 K
β = 92.72 (3)°	Needle, colourless
*V* = 779.6 (3) Å^3^	0.40 × 0.05 × 0.05 mm
*Z* = 4	

### Data collection

**Table:**

Rigaku Mercury2 diffractometer	1773 independent reflections
Radiation source: fine-focus sealed tube	1581 reflections with *I* > 2σ(*I*)
graphite	*R*_int_ = 0.034
Detector resolution: 13.6612 pixels mm^-1^	θ_max_ = 27.5°, θ_min_ = 2.6°
CCD profile fitting scans	*h* = −7→7
Absorption correction: multi-scan (*CrystalClear*; Rigaku, 2005)	*k* = −20→20
*T*_min_ = 0.65, *T*_max_ = 0.77	*l* = −10→11
3848 measured reflections	

### Refinement

**Table:**

Refinement on *F*^2^	Hydrogen site location: inferred from neighbouring sites
Least-squares matrix: full	H-atom parameters constrained
*R*[*F*^2^ > 2σ(*F*^2^)] = 0.025	*w* = 1/[σ^2^(*F*_o_^2^) + (0.0143*P*)^2^] where *P* = (*F*_o_^2^ + 2*F*_c_^2^)/3
*wR*(*F*^2^) = 0.050	(Δ/σ)_max_ < 0.001
*S* = 0.87	Δρ_max_ = 0.47 e Å^−^^3^
1773 reflections	Δρ_min_ = −0.41 e Å^−^^3^
93 parameters	Extinction correction: *SHELXTL* (Version 5.1; Sheldrick, 2008), Fc^*^=kFc[1+0.001xFc^2^λ^3^/sin(2θ)]^-1/4^
2 restraints	Extinction coefficient: 0.0206 (8)
Primary atom site location: structure-invariant direct methods	Absolute structure: Flack (1983), 872 Friedels pairs
Secondary atom site location: difference Fourier map	Flack parameter: 0.004 (13)

### Special details

**Table:**

Geometry. All esds (except the esd in the dihedral angle between two l.s. planes) are estimated using the full covariance matrix. The cell esds are taken into account individually in the estimation of esds in distances, angles and torsion angles; correlations between esds in cell parameters are only used when they are defined by crystal symmetry. An approximate (isotropic) treatment of cell esds is used for estimating esds involving l.s. planes.
Refinement. Refinement of F^2^ against ALL reflections. The weighted R-factor wR and goodness of fit S are based on F^2^, conventional R-factors R are based on F, with F set to zero for negative F^2^. The threshold expression of F^2^ > 2sigma(F^2^) is used only for calculating R-factors(gt) etc. and is not relevant to the choice of reflections for refinement. R-factors based on F^2^ are statistically about twice as large as those based on F, and R- factors based on ALL data will be even larger.

### Fractional atomic coordinates and isotropic or equivalent isotropic displacement parameters (Å^2^)

**Table:**

	*x*	*y*	*z*	*U*_iso_*/*U*_eq_	
Br1	0.05750 (10)	0.208462 (17)	0.44566 (9)	0.04064 (12)	
N1	0.0515 (5)	0.32177 (17)	0.7619 (3)	0.0347 (6)	
H1A	0.0332	0.2950	0.6701	0.052*	
H1B	−0.0713	0.3127	0.8188	0.052*	
H1C	0.1780	0.3027	0.8140	0.052*	
C6	−0.0799 (6)	0.4675 (3)	0.7945 (5)	0.0372 (10)	
H6	−0.2028	0.4473	0.8503	0.045*	
C3	0.2867 (8)	0.5276 (3)	0.6309 (5)	0.0451 (11)	
H3	0.4102	0.5483	0.5762	0.054*	
C1	0.0755 (6)	0.4115 (2)	0.7337 (5)	0.0309 (9)	
C4	0.1322 (8)	0.5822 (3)	0.6917 (5)	0.0500 (12)	
H4	0.1516	0.6399	0.6788	0.060*	
C7	0.4233 (6)	0.3856 (2)	0.5783 (4)	0.0410 (8)	
N2	0.5521 (6)	0.3440 (2)	0.5163 (4)	0.0618 (10)	
C2	0.2611 (6)	0.4411 (3)	0.6501 (4)	0.0346 (9)	
C5	−0.0547 (7)	0.5521 (3)	0.7733 (5)	0.0474 (12)	
H5	−0.1613	0.5894	0.8130	0.057*	

### Atomic displacement parameters (Å^2^)

**Table:**

	*U*^11^	*U*^22^	*U*^33^	*U*^12^	*U*^13^	*U*^23^
Br1	0.03349 (17)	0.04202 (18)	0.04729 (19)	−0.0034 (2)	0.01111 (12)	−0.0112 (3)
N1	0.0284 (14)	0.0369 (15)	0.0396 (16)	−0.0027 (12)	0.0104 (12)	−0.0036 (13)
C6	0.031 (2)	0.043 (2)	0.038 (2)	−0.0023 (18)	0.0075 (17)	−0.0038 (18)
C3	0.044 (3)	0.046 (3)	0.046 (3)	−0.012 (2)	0.007 (2)	0.003 (2)
C1	0.027 (2)	0.035 (2)	0.0309 (18)	0.0005 (15)	0.0021 (15)	−0.0028 (15)
C4	0.065 (3)	0.031 (2)	0.055 (3)	−0.004 (2)	0.008 (2)	0.003 (2)
C7	0.0303 (18)	0.049 (2)	0.044 (2)	−0.0078 (17)	0.0101 (16)	0.0045 (18)
N2	0.049 (2)	0.070 (2)	0.069 (2)	0.0068 (19)	0.031 (2)	0.0025 (18)
C2	0.032 (2)	0.039 (2)	0.032 (2)	−0.0017 (17)	0.0039 (17)	0.0032 (16)
C5	0.056 (3)	0.041 (2)	0.045 (3)	0.012 (2)	0.006 (2)	−0.007 (2)

### Geometric parameters (Å, °)

**Table:**

N1—C1	1.455 (5)	C3—C2	1.393 (6)
N1—H1A	0.8900	C3—H3	0.9300
N1—H1B	0.8900	C1—C2	1.396 (5)
N1—H1C	0.8900	C4—C5	1.396 (6)
C6—C5	1.366 (6)	C4—H4	0.9300
C6—C1	1.382 (5)	C7—N2	1.142 (4)
C6—H6	0.9300	C7—C2	1.444 (6)
C3—C4	1.365 (6)	C5—H5	0.9300
			
C1—N1—H1A	109.5	C6—C1—N1	120.1 (3)
C1—N1—H1B	109.5	C2—C1—N1	119.7 (3)
H1A—N1—H1B	109.5	C3—C4—C5	120.4 (4)
C1—N1—H1C	109.5	C3—C4—H4	119.8
H1A—N1—H1C	109.5	C5—C4—H4	119.8
H1B—N1—H1C	109.5	N2—C7—C2	177.0 (4)
C5—C6—C1	120.6 (4)	C3—C2—C1	118.7 (4)
C5—C6—H6	119.7	C3—C2—C7	118.6 (4)
C1—C6—H6	119.7	C1—C2—C7	122.6 (4)
C4—C3—C2	120.5 (4)	C6—C5—C4	119.5 (4)
C4—C3—H3	119.7	C6—C5—H5	120.2
C2—C3—H3	119.7	C4—C5—H5	120.2
C6—C1—C2	120.2 (4)		
			
C5—C6—C1—C2	0.2 (6)	N1—C1—C2—C3	−177.0 (4)
C5—C6—C1—N1	177.8 (4)	C6—C1—C2—C7	−177.4 (4)
C2—C3—C4—C5	−0.4 (7)	N1—C1—C2—C7	5.0 (6)
C4—C3—C2—C1	−0.5 (6)	C1—C6—C5—C4	−1.1 (7)
C4—C3—C2—C7	177.6 (4)	C3—C4—C5—C6	1.2 (7)
C6—C1—C2—C3	0.6 (6)		

### Hydrogen-bond geometry (Å, °)

**Table:**

*D*—H···*A*	*D*—H	H···*A*	*D*···*A*	*D*—H···*A*
N1—H1A···Br1	0.89	2.36	3.234 (3)	168
N1—H1B···Br1^i^	0.89	2.47	3.355 (3)	173
N1—H1C···Br1^ii^	0.89	2.42	3.286 (3)	164

Symmetry codes: (i) *x*−1/2, −*y*+1/2, *z*+1/2; (ii) *x*+1/2, −*y*+1/2, *z*+1/2.
